# What Can Interaction Webs Tell Us About Species Roles?

**DOI:** 10.1371/journal.pcbi.1004330

**Published:** 2015-07-21

**Authors:** Elizabeth L. Sander, J. Timothy Wootton, Stefano Allesina

**Affiliations:** 1 Department of Ecology & Evolution, University of Chicago, Chicago, Illinois, United States of America; 2 Computation Institute, University of Chicago, Chicago, Illinois, United States of America; University of North Carolina at Wilmington, UNITED STATES

## Abstract

The group model is a useful tool to understand broad-scale patterns of interaction in a network, but it has previously been limited in use to food webs, which contain only predator-prey interactions. Natural populations interact with each other in a variety of ways and, although most published ecological networks only include information about a single interaction type (*e.g.*, feeding, pollination), ecologists are beginning to consider networks which combine multiple interaction types. Here we extend the group model to signed directed networks such as ecological interaction webs. As a specific application of this method, we examine the effects of including or excluding specific interaction types on our understanding of species roles in ecological networks. We consider all three currently available interaction webs, two of which are extended plant-mutualist networks with herbivores and parasitoids added, and one of which is an extended intertidal food web with interactions of all possible sign structures (+/+, -/0, etc.). Species in the extended food web grouped similarly with all interactions, only trophic links, and only nontrophic links. However, removing mutualism or herbivory had a much larger effect in the extended plant-pollinator webs. Species removal even affected groups that were not directly connected to those that were removed, as we found by excluding a small number of parasitoids. These results suggest that including additional species in the network provides far more information than additional interactions for this aspect of network structure. Our methods provide a useful framework for simplifying networks to their essential structure, allowing us to identify generalities in network structure and better understand the roles species play in their communities.

## Author Summary

Ecological interactions are highly diverse even when considering a single species: the species might feed on a first, disperse the seeds of a second, and pollinate a third. Here we extend the group model, a method for identifying broad patterns of interaction across a food web, to networks which contain multiple types of interactions. Using this new method, we ask whether the traditional approach of building a network for each type of interaction (food webs for consumption, pollination webs, seed-dispersal webs, host-parasite webs) can be improved by merging all interaction types in a single network. In particular, we test whether combining different interaction types leads to a better definition of the roles species play in ecological communities. We find that, although having more information necessarily leads to better results, the improvement is only incremental if the linked species remain unchanged. However, including a new interaction type that attaches new species to the network substantially improves performance. This method provides insight into possible implications of merging different types of interactions and allows for the study of coarse-grained structure in any signed network, including ecological interaction webs, gene regulation networks, and social networks.

## Introduction

Networks are a useful tool to understand patterns of interactions in an ecological community. As ecologists have collected more and more network data, the size of published networks has grown dramatically, with many networks now containing hundreds of species. To make sense of these increasingly complex data, we need tools to simplify the network down to its essential structure, allowing us to identify general patterns of interaction in the community.

The group model (equivalent to the stochastic block model from the social science literature, [1]) is a useful way to simplify and understand ecological networks. It has previously been common to characterize species in terms of their ecological niches, that is, by the resources or predators of a given species. Species with identical niches were considered “trophic species”, and ecological networks were often simplified by combining them [2]. However, this approach is highly sensitive to small changes or errors in the network structure, since a single missing or false interaction can change which species may be combined. The group model [3] models the concept of *ecological equivalence* [4] (distinct from the term as used in neutral theory). Species are considered to be ecologically equivalent if their predators and prey are equivalent, who are equivalent if their predators and prey are equivalent, and so on. In other words, species are grouped together if they eat and are eaten by the same other groups. This recursive definition implies that species which are far from each other in the network may still impact each other’s grouping. This reflects the ecological reality of the complex ways in which species in a network influence each others’ dynamics, for example, via trophic cascades or apparent competition [5, 6]. Since ecologically equivalent species prey on and are preyed on by the same other groups, species within a group can be thought of as filling the same role in the community, and may be expected to operate in the community in similar ways. The group structure is also able to capture both modular (compartmental) and anti-modular (*i.e*., trophic levels) aspects of the network, both of which are found in ecological networks. Thus, the group model is a useful way to gain a coarse-grained view of ecological dynamics and the niches that are filled in the community.

A limitation of the group model is the fact that it can only group species based on a single interaction type (usually predator-prey interactions, although it could in principle be applied to any one interaction type). Of course, species in ecological communities interact in diverse ways, and different interaction types operate simultaneously to influence community dynamics [7, 8]. Although ecologists have traditionally built separate networks for each interaction type, such as food webs (containing only feeding interactions), or plant-pollinator and plant-seed-disperser networks (containing only mutualistic interactions) [9–14], there is a growing recognition that different interaction types may work in concert to influence communities. Both empirical and simulation studies have demonstrated the complex ways in which mutualisms and antagonisms may interact [7, 15, 16]. For example, recent work has begun to explore the possible effects of including cheaters in mutualistic networks [15, 17, 18], modelling communities with multiple interaction types [19, 20], and combining mutualistic networks and food webs [21–23].

**Fig 1:**
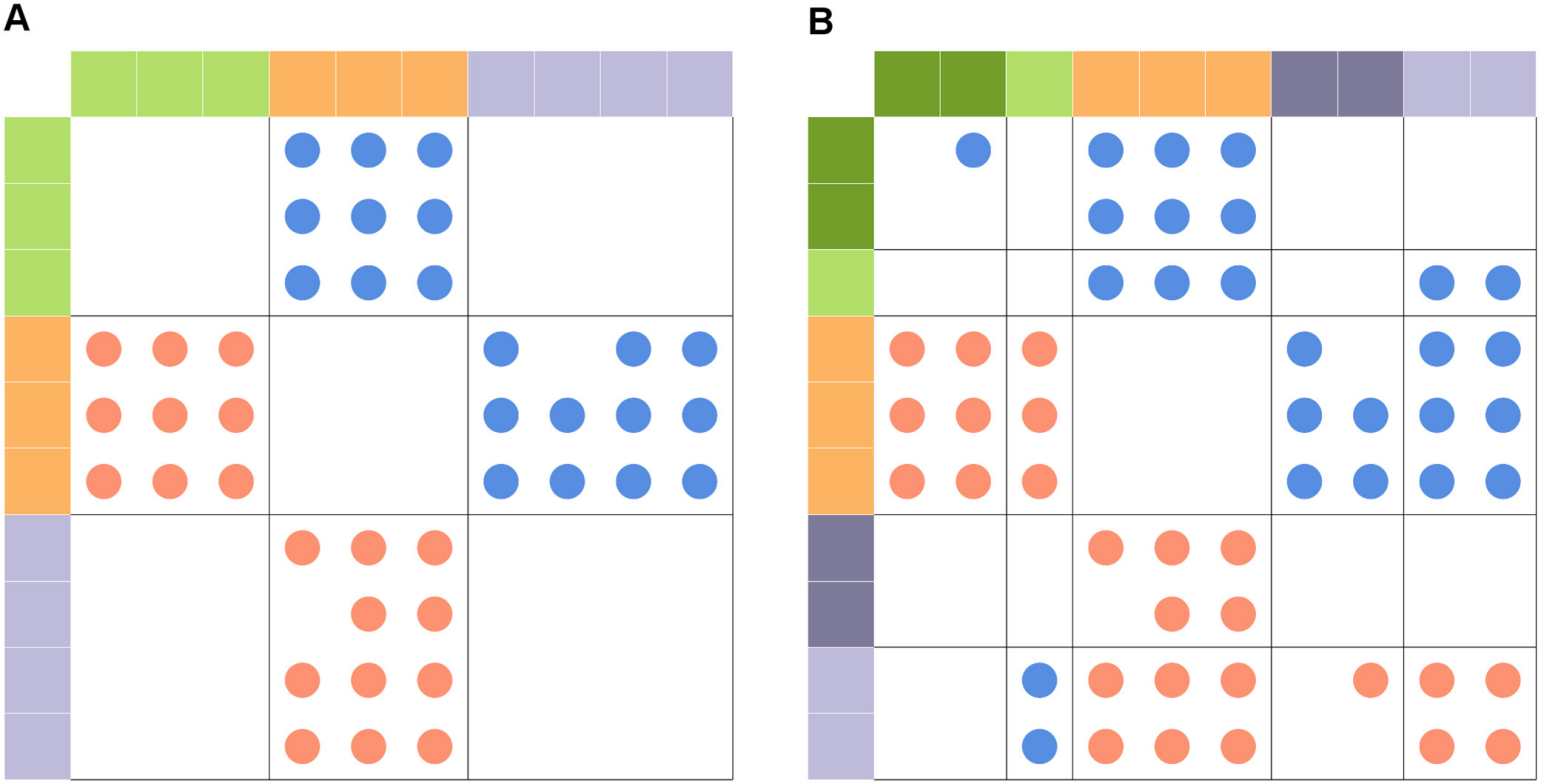
Example 10-species network partitioned using the group model. Each row and column represents a species, and each dot in the heatmap represents an interaction between two species (red for negative impact of column on row, blue for positive impact of column on row, white for no interaction). Colors on the outer edge correspond to group membership. In (A), only trophic links are included, and the network is partitioned into 3 groups. In (B), both trophic and nontrophic interactions are included. The mutualism between the light purple and light green groups has caused the green and purple groups from part (A) to split into two subgroups. In this example, nontrophic interactions serve to refine trophic groups into subgroups, but additional interactions could potentially reinforce or directly conflict with groupings based on a single interaction type.

Here, we extend the group model from unsigned (single interaction type) to signed directed adjacency matrices, allowing ecologists to study the general structure of merged interaction networks. Using this extension of the group model, species in a group tend to interact with other groups *in the same way*. We demonstrate one possible use of this method by considering how including or excluding different interaction types changes our understanding of group structure in three interaction webs (the only three such networks currently available). Despite the growing body of work on potential impacts of merging networks with multiple interaction types, it is unknown whether these merged networks provide new, meaningful information about species roles at the network level. While it is intuitive that more types of interaction data would provide more (or more accurate) information about the roles species play in their communities, it is valuable to study this question directly. Clearly, species groupings will be contingent on the species and interactions that are included in the network. Adding interactions may reinforce, refine, or contradict the previous understanding of species roles (Fig 1). We study how our understanding of species roles changes based on different types using three networks of two types. The Tatoosh mussel bed network is an intertidal food web with additional interaction types included. This network contains feeding (+/-), competitive (-/-), mutualist (+/+), commensal (+/0), and amensal (-/0) interactions. For this network, we compare how species group based on all interaction types, only trophic interactions, and only nontrophic interactions. The other interaction networks, from Doñana Biological Reserve [24] and Norwood farm [25], are terrestrial networks which include plants, plant mutualists, plant herbivores, and in the Norwood web, parasitoids which parasitize herbivores. These networks are structurally different from the Tatoosh web in that they are almost entirely multipartite; that is, they are composed of “layers” of species which only interact with the layers above and below (*i.e*., mutualists interact with plants, plants interact with mutualists and herbivores, and herbivores interact with plants and parasitoids). In these networks, only plants are involved in both feeding and mutualistic interactions, so we consider how the grouping of plants is affected by including mutualists, herbivores, or both. For the Norwood web, we also consider the effect of including or excluding parasitoids on plant groupings. Using this framework, we study if and how omitting specific interaction types changes our understanding of network structure and species roles.

## Methods

### Ethics Statement

The Makah Tribal Council has granted permission to the Wootton lab for access to Tatoosh Island.

### Signed Interaction Networks

A food web composed of *S* species may be represented by an adjacency matrix *A*, where *A*_*ij*_ is 1 if *j* consumes *i*, and 0 otherwise. Similarly, interaction networks may be represented by a signed adjacency matrix where *A*_*ij*_ is 1 if the growth rate of *j* positively depends on the presence of *i*, -1 if its growth rate negatively depends on *i*, and 0 otherwise. Such a matrix may be thought of as containing the signs of the community matrix (the Jacobian evaluated at equilibrium), as opposed to a matrix of zero-sum energy or nutrient flow throughout the system (*sensu* [26]). Some interactions, such as competition for carbon or another nutrient, may be considered an indirect interaction which is the product of two consumer-resource interactions (two direct consumer-carbon interactions in this case). In this example, carbon would be incorporated into the differential equations underlying the community matrix.

Since we are interested in how species group within an interconnected network, we require that the complete interaction networks are a single weakly connected component (that is, isolated subgraphs were removed).

### Network Data

Interaction data for Tatoosh Island were collected from the intertidal middle zone based on observed interactions and natural history information. This middle zone on Tatoosh is dominated by the mussel *Mytilus californianus*. This mussel-dominated band is defined from below by the presence of *Pisaster ochraceus*, which consumes *M. californianus* [27], and from above by physiological constraints, such as time spent submerged [28]. The signed interaction network contains 110 taxa and 1898 interaction pairs (869 +/-, 5 +/+, 208 +/0, 492 -/0, and 324 -/-). This dataset is available on Dryad (DOI:10.5061/dryad.39jv1)

The largest weakly connected component was taken from Doñana Biological Reserve and Norwood Farm (data made available in [24] and [25], respectively). The Doñana network contains 391 species total, with 170 plants, 207 mutualists (576 mutualistic interactions), and 14 herbivores (221 feeding interactions). The Norwood network contains 445 species, with 91 plants, 251 mutualists (569 mutualistic interactions), 62 herbivores (570 herbivory interactions), and 43 parasitoids (367 parasitic interactions). Two species were classified in two categories: one which interacted both as a mutualist and as an herbivore, and another as both a mutualist and a parasitoid.

Because taxonomically similar species are generally expected to fill similar roles in a community [29] (but see [30]), taxonomic data provide a potential natural grouping. Tatoosh taxa were classified to kingdom and phylum, and plants in the Doñana and Norwood webs were classified to the order level. Taxonomic levels were chosen to have a number of groups that was similar to the number of groups found by the group model for the complete networks. The high phylogenetic diversity of the Tatoosh system meant that taxonomic groupings beyond the phylum level included too many groups to provide useful information about the system. Taxonomic data for all three networks were gathered from the Integrated Taxonomic Information System (ITIS) database and Encyclopedia of Life (see S1 Text for details).

### Group Model for Signed Directed Graphs

Consider an interaction web with *S* species and *L* links, *K* of which are positive and *L* − *K* negative. The data can be represented using a signed directed adjacency matrix *N*. What is the probability of obtaining *N* by chance? A simple model of random signed network structure is similar to an Erdős-Rényi random graph with *S* species and a fixed probability *c* of connecting any two nodes, with an additional probability *π* that a link is positive. Then the probability of obtaining exactly *N* using this model is:

$$P(N(S,L,K)|c,\pi )=c^{L}\pi^{K}{\left(1-c\right)}^{Z}{\left(1-\pi \right)}^{L-K}$$ (1)

where *Z* = *S* ^2^ − *L* is the number of zeros in the matrix. This likelihood is maximized when $\hat{c}=\frac{L}{L+Z}$ and $\hat{\pi }=\frac{K}{L}$.

Now to see this in the context of the group model, consider N when divided into two groups, *X* and *Y*. If *N* is a mutualistic web, these groups might correspond to plants and pollinators. Now the random network process involves eight probabilities: *c*_*xx*_, the probability of a species in group *X* connecting to another species in group *X*, *π*_*xx*_, the probability of a link between two species in *X* being positive, *c*_*xy*_, the probability of a species in *X* connecting to a species in *Y*, and so on for *c*_*yx*_, *c*_*yy*_, *π*_*yy*_, *π*_*xy*_, and *π*_*yx*_, which are defined similarly. Note that *c*_*xy*_ and *c*_*yx*_ are not necessarily equal (nor are *π*_*xy*_ and *π*_*yx*_), since *N* need not be symmetric. Then the probability of obtaining *N* given the two groups is:

$$\begin{array}{c} P(N\left(S,L,K\right)|c_{ij},\pi_{ij},i,j\in x,y)=\prod_{i\in (X,Y)}\prod_{j\in (X,Y)}c_{ij}^{L_{ij}}\pi_{ij}^{K_{ij}}{\left(1-c_{ij}\right)}^{Z_{ij}}{\left(1-\pi_{ij}\right)}^{L_{ij}-K_{ij}} \end{array}$$ (2)

Analagous to Eq 1, this likelihood is maximized when ${\hat{c}}_{ij}=\frac{L_{ij}}{L_{ij}+Z_{ij}}$ and ${\hat{\pi }}_{ij}=\frac{K_{ij}}{L_{ij}}$ for all combinations of groups. This can be generalized to *g* groups as follows:

$$\begin{array}{c} P(N\left(S,L,K\right)|c_{ij},\pi_{ij},i,j\in 1\;:\;g)=\prod_{i=1}^{g}\prod_{j=1}^{g}c_{ij}^{L_{ij}}\pi_{ij}^{K_{ij}}{\left(1-c_{ij}\right)}^{Z_{ij}}{\left(1-\pi_{ij}\right)}^{L_{ij}-K_{ij}} \end{array}$$ (3)

When *g* = 1, this is equivalent to Eq 1. When *g* = *S*, each species is in its own group, and the likelihood is 1. Such a grouping is not very informative, so we need to perform model selection. Using a uniform prior (such that the probability of each model is $\frac{1}{2}$), it is possible to analytically calculate a Bayes factor to compare two groupings. For groupings *G*_1_ and *G*_2_, the Bayes factor is given by:

$$B=\frac{P(N|G_{1})}{P(N|G_{2})}$$ (4)

where *P*(*N*∣*G*_*i*_) is the marginal likelihood

$$\begin{array}{c} \int_{0}^{1}\cdots \int_{0}^{1}P\;(c_{ij},\pi_{ij},i,j\in 1\;:\;S|G_{i})\;P\;(N|c_{ij},\pi_{ij},i,j\in 1\;:\;g,G_{i})\;dc_{11}\ldots dc_{gg}d\pi_{11}\ldots d\pi_{gg} \end{array}$$ (5)

which can be analytically integrated to give:

$$\prod_{i=1}^{g}\prod_{j=1}^{g}\frac{K_{ij}!Z_{ij}!\left(L_{ij}-K_{ij}\right)!}{\left(1+L_{ij}\right)\left(1+L_{ij}+Z_{ij}\right)!}$$ (6)

Because there are many possible groupings to choose from, we compared the marginal likelihoods of the groupings when searching for the best grouping, rather than explicitly calculating *B* for each pair.

We searched for the optimal grouping using Metropolis-Coupled Markov Chain Monte Carlo (*MC* ^3^) with a Gibbs sampler (see S1 Text). It is not feasible to exhaustively search the space of all possible groupings, so the best groupings found are not guaranteed to be the optimal ones, but for simplicity, we refer to them as “best groupings” throughout.

### Partition Similarity

The entropy of a partition *A* is an information theoretic measure of the information content or uncertainty of that partition, measured in nats [31]. A partition where all species are in the same group would have low entropy, because we can be quite certain of which group any given species belongs to. In contrast, a partition with many groups would have higher entropy, since it is difficult to make an *a priori* guess about the group identity of a given species. Entropy is calculated as:

$$H\left(A\right)=-\sum_{a\in A}p\left(a\right)\ln(p(a))$$ (7)

This metric is known as Shannon entropy, commonly used in ecology to measure the diversity of a community [32]. The joint entropy of two partitions *A* and *B* is similarly defined:

$$H\left(A,B\right)=-\sum_{a\in A}\sum_{b\in B}p\left(a,b\right)\ln(p(a,b))$$ (8)

This can be thought of as the union between *H*(*A*) and *H*(*B*), since it sums over all joint probabilities of the two entropies. Note that for all entropies, 0 ln(0) is given to be 0, so that including values with probability zero does not change the entropy [31].

To measure the similarity between two partitions, we then wish to know how much entropy the partitions share. This is known as the mutual information (*MI*), which quantifies the reduction in entropy of partition *B* when partition *A* is known. It is calculated as

$$MI_{AB}=H\left(A\right)+H\left(B\right)-H\left(A,B\right)$$ (9)

This can be thought of as the intersection between *H*(*A*) and *H*(*B*). Converting this measure into probabilities gives us

$$MI_{AB}=-\sum_{a\in A}p\left(a\right)\ln(p(a))-\sum_{b\in B}p\left(b\right)\ln(p(b))+\sum_{a\in A}\sum_{b\in B}p\left(a,b\right)\ln(p(a,b))$$ (10)

$$=-\sum_{a\in A}\sum_{b\in B}p\left(a,b\right)\ln(p(a))-\sum_{a\in A}\sum_{b\in B}p\left(a,b\right)\ln(p(b))+\sum_{a\in A}\sum_{b\in B}p\left(a,b\right)\ln(p(a,b))$$ (11)

$$=\sum_{a\in A}\sum_{b\in B}p\left(a,b\right)\ln(\frac{p(a,b)}{p(a)p(b)})$$ (12)

To see how this is calculated for a partition generated by the group model, see Box 1.

**Box 1. Calculation of *MI* for ecological partitions**

Consider the following two partitions for a five-species grouping:

$$\begin{array}{cc} & \text{Partition}\;A:\;1\;2\;1\;2\;1\\ & \text{Partition}\;B:\;\alpha \;\beta \;\gamma \;\beta \;\beta \end{array}$$

where each column is a species, and numbers and Greek letters correspond to group identity within partitions *A* and *B*, respectively. Using these groupings, we can create a joint count matrix:

$$\begin{array}{cccc} & 1 & 2 & n_{i\cdot }\\ \alpha & 1 & 0 & 1\\ \beta & 1 & 2 & 3\\ \gamma & 1 & 0 & 1\\ n_{\cdot j} & 3 & 2 & 5 \end{array}$$

where each table entry *n*_*ij*_ is the number of species which are in group *i* in partition *A* and in group *j* in partition *B*. The row totals *n*_*i*⋅_ and column totals *n*_⋅*j*_ are the marginal counts, *i.e*., the total number of species in group *i* in partition *A* or the total number of species in group *j* in partition *B*, respectively. These counts can easily be converted into probabilities by dividing by the total number of species *N* (in this case, 5). Then $p(a)=\frac{n_{a\cdot }}{N}$, $p(b)=\frac{n_{\cdot b}}{N}$, and $p(a,b)=\frac{n_{ab}}{N}$. This gives us

$$MI_{AB}=\sum_{i=1}^{g_{A}}\sum_{j=1}^{g_{B}}\frac{n_{ij}}{N}\ln(\frac{n_{ij}}{N}\frac{1}{\frac{n_{i\cdot }}{N}}\frac{1}{\frac{n_{\cdot j}}{N}})$$ (13)

$$=\sum_{i=1}^{g_{A}}\sum_{j=1}^{g_{B}}\frac{n_{ij}}{N}\ln(\frac{n_{ij}N}{n_{i\cdot }n_{\cdot j}})$$ (14)

for our example:

$$MI_{AB}=\frac{1}{5}\ln(\frac{1\cdot 5}{1\cdot 3})+\cdots +\frac{0}{5}\ln(\frac{0\cdot 5}{1\cdot 2})\approx .102$$ (15)

**Fig 2:**
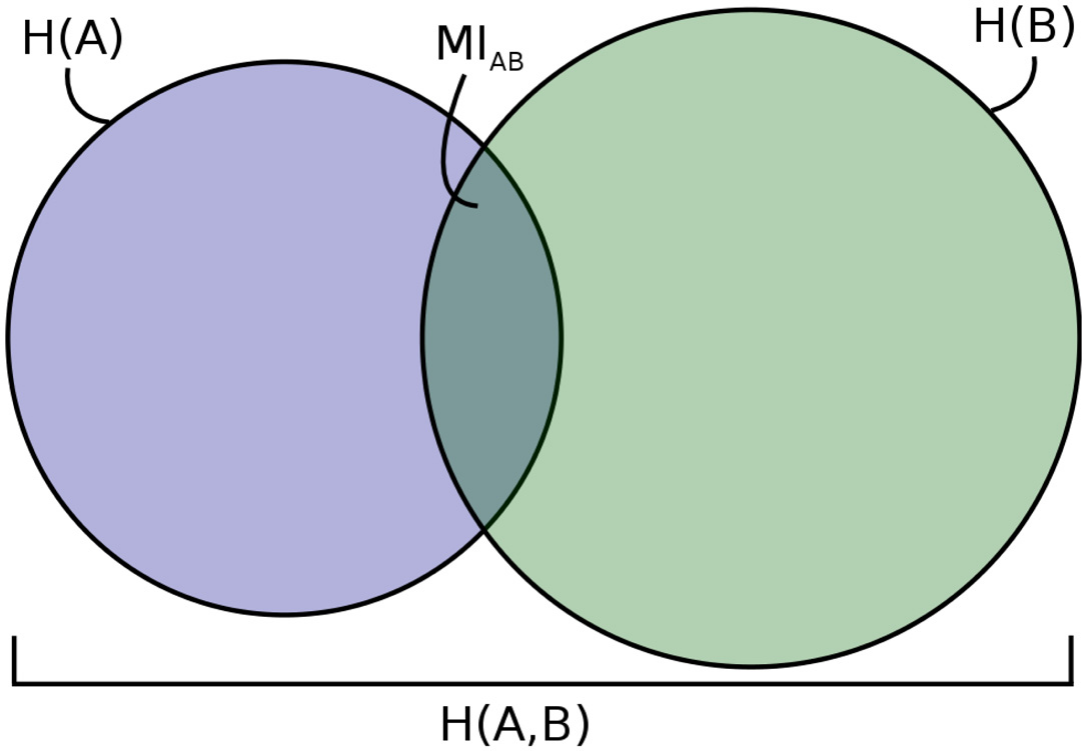
Mutual Information Venn Diagram for 5-species partitions *A* and *B*. Left circle represents *H*(*A*), right circle represents *H*(*B*), and the intersection represents *MI*_*AB*_. All areas are proportional to the values they represent.

Because the *MI* is the shared entropy between two partitions, it can be represented as a Venn Diagram, with circle areas proportional to *H*(*A*) and *H*(*B*), and the area of overlap between the circles proportional to the mutual information. The corresponding diagram for our example is given in Fig 2, with *H*(*A*) = .673, *H*(*B*) = .950, and *MI*_*AB*_ = .102.

Significance of *MI* values was estimated based on a randomization test. To estimate how likely it was to get an equal or higher *MI* by chance, each of the two partitions were shuffled, such that the randomized partitions conserved the number of species in each group (and therefore the upper bound on the *MI*, see S1 text for details), but not their identities. The *MI* was then calculated for the randomized partitions. This process was repeated one million times, and the *p*-value was estimated as the probability of getting an *MI* greater than or equal to the observed *MI* for the two partitions. Since the probability of getting a given *MI* is based both on the entropies and the groupings, it is possible to get a low *p*-value for a relatively low *MI*, or a high *p*-value for a high *MI*. Code for calculating partition similarity, obtaining taxonomic data, and running the search algorithm are available on GitHub at https://github.com/esander91/SignedGroupModel.

## Results

### Tatoosh Island

**Fig 3:**
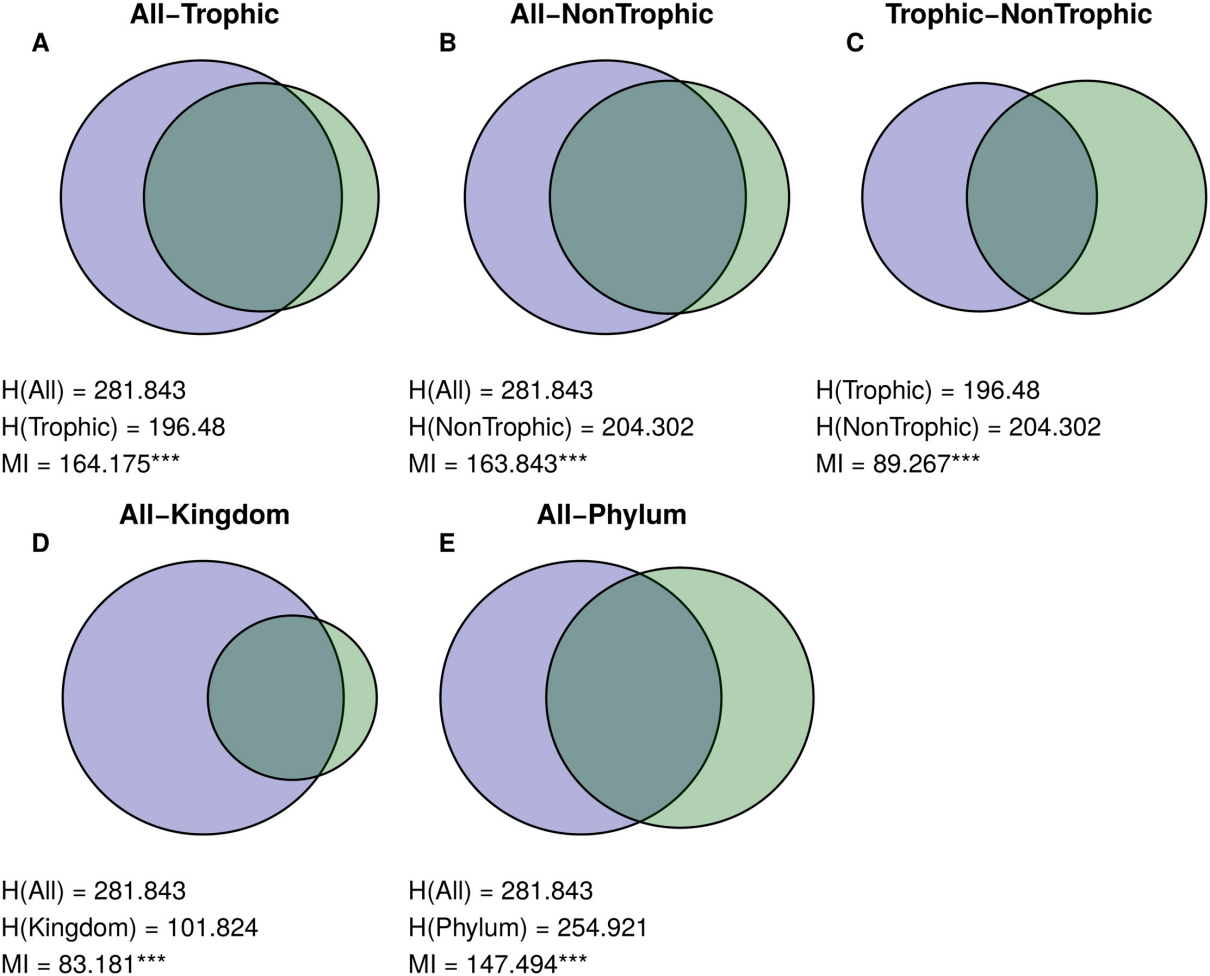
Similarities between Tatoosh Mussel Bed partitions. Venn Diagrams showing the similarity between pairs of partitions in the Tatoosh Mussel Bed: (A) the complete and trophic networks, (B) complete and nontrophic networks, (C) trophic and nontrophic, and complete and taxonomic groupings (D and E). Venn Diagrams are structured as in Fig 2, where the size of the left circle is proportional in area to the entropy of the first partition listed (*H*(*A*)), the right circle’s area represents the entropy of the second partition listed (*H*(*B*)), and the overlap between the circles is proportional to the Mutual Information values (*MI*). Stars next to *MI* values denote significance level (* <.05, ** <.01, *** <.001). Note that this figure includes only the partition comparisons that are discussed in the main text. For all partition comparisons, see S3 Fig.

**Fig 4:**
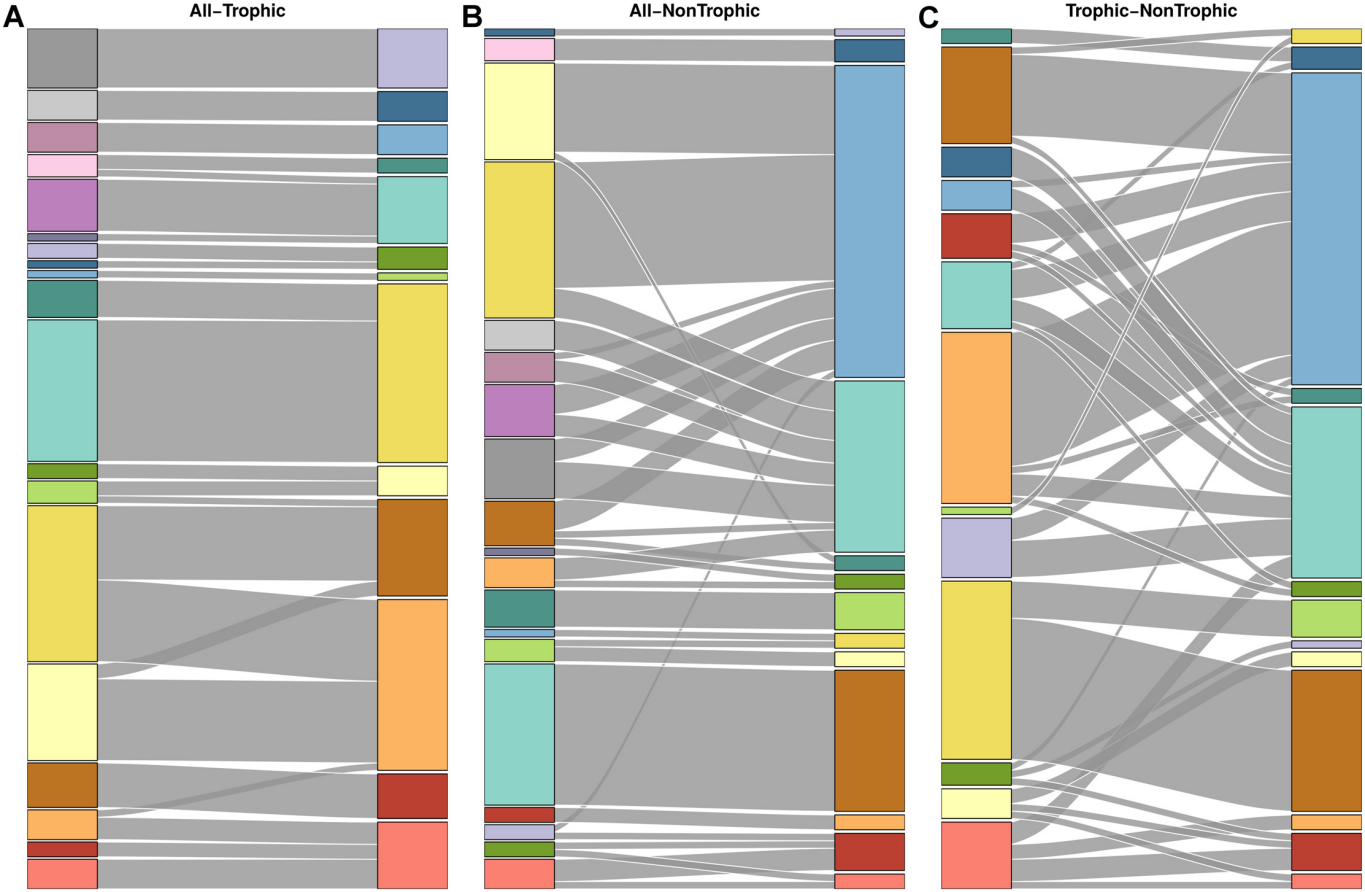
Similarity between Tatoosh network groupings. Alluvial diagrams comparing the species groupings for (A) complete and trophic webs, (B) complete and nontrophic webs, and (C) trophic and nontrophic webs. Complete network coloring matches colors in Fig 5. Note that the light red group in the complete grouping does not necessarily correspond to the light red group in the trophic group, and so on. Flows between groupings show species in common between two groups; line thickness is proportional to number of species in common. The complete Tatoosh network is organized into groups that are almost perfectly nested in the trophic grouping. The complete grouping also matches closely with the nontrophic groupings, but the trophic and nontrophic groupings are comparatively dissimilar.

**Fig 5:**
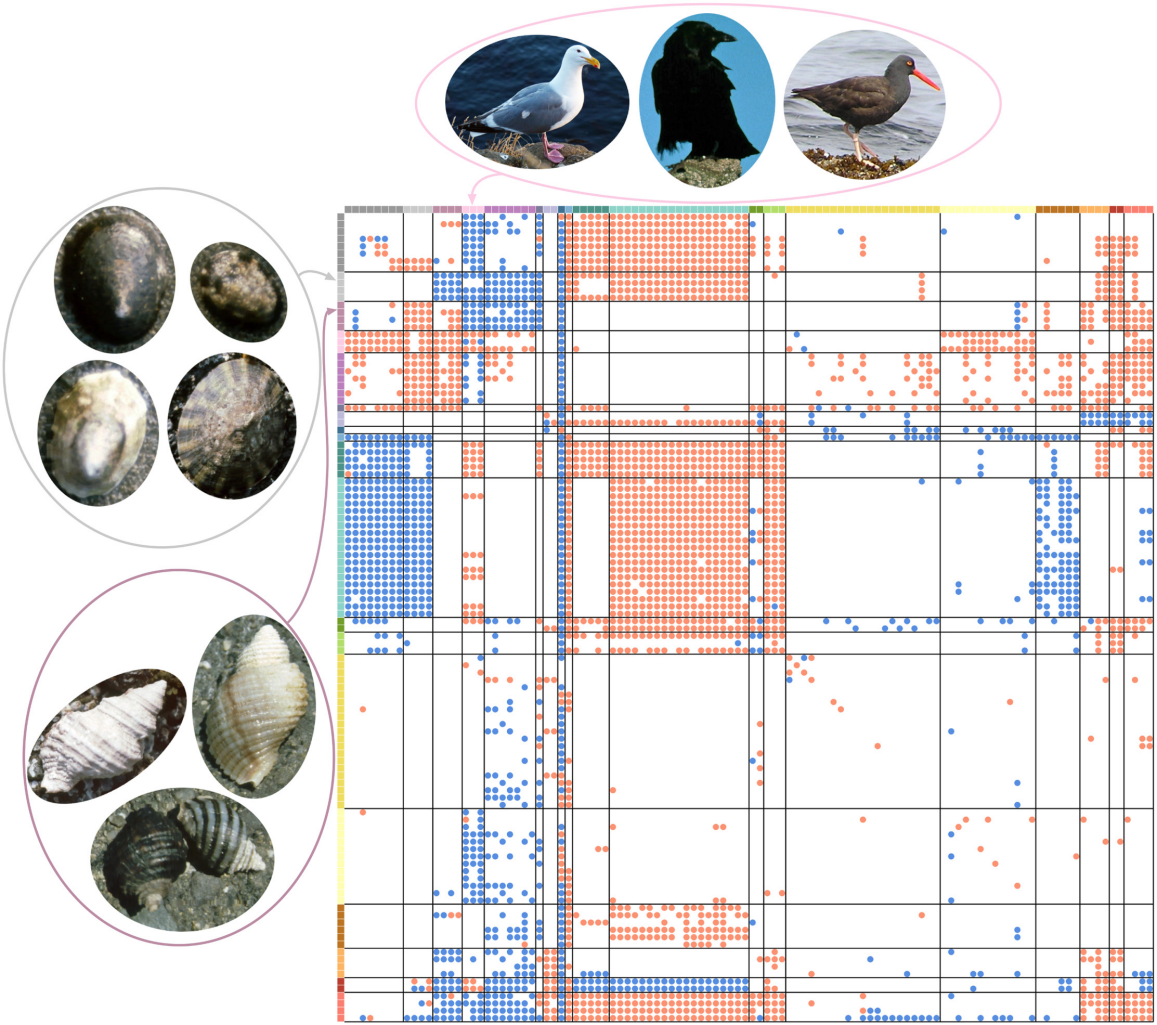
Matrix structure of complete Tatoosh network, organized by groups. The best complete Tatoosh network grouping, displayed in matrix form. Dot colors in the top row and leftmost column represent group identity (19 groups total). Red and blue dots in the matrix are defined as in Fig 1. Many of the groups in the partition correspond closely to *a priori* ecological knowledge about the system, for example in the foraging birds (dusty purple), limpets (light blue), and predatory snails (dark aqua). This highlights the success of this method in identifying relevant groups, even in the absence of specific ecological information. Full list of species and their group identities given in S1 Table.

Both the partitions for the network with all interactions and the network with trophic interactions grouped species in a similar way (Fig 3). Though the complete web grouping divided taxa into more groups than the trophic grouping did (19 and 13 groups, respectively), many of these additional groups were simply nested within groups from the trophic one (Fig 4A). Many groups corresponded strikingly well to known ecologically relevant groups in this community, including predatory snails (*n* = 4), kelps (*n* = 5), limpets (*n* = 4), and foraging birds (*n* = 3; Fig 5).

The complete grouping was also quite similar to the nontrophic grouping. In contrast to the trophic partition, which captured the general structure of the complete grouping across the entire web (S1 Fig), the nontrophic partition captured portions of the complete one very precisely, but grouped many species into one of two broad groups. Although the nontrophic network contained more interactions than the trophic one overall, these interactions were unevenly spread across species; in particular, sessile species tended to competitively interact with other species, while mobile species often only interacted with a few other species in a nontrophic fashion. As a result, many sessile species (particularly algae and barnacles; see S2 Fig and S1 Table) were organized into similar groups as in the complete grouping, while most other species were placed into one of two large groups which were sparsely connected to the rest of the network. The trophic and nontrophic groupings were less similar to each other than to the complete grouping (Fig 4C), but were much more similar to each other than expected by chance. Jackknife resampling of the complete network showed that group structure was robust to removal of individual species, as measured by ratio between the *MI* for the Jackknifed and original groupings and the maximum *MI* possible given their entropies (mean $\frac{MI}{MI_{MAX}}=.99,\sigma =.014$; see S1 Text for methodological details).

### Doñana Biological Reserve

**Fig 6:**
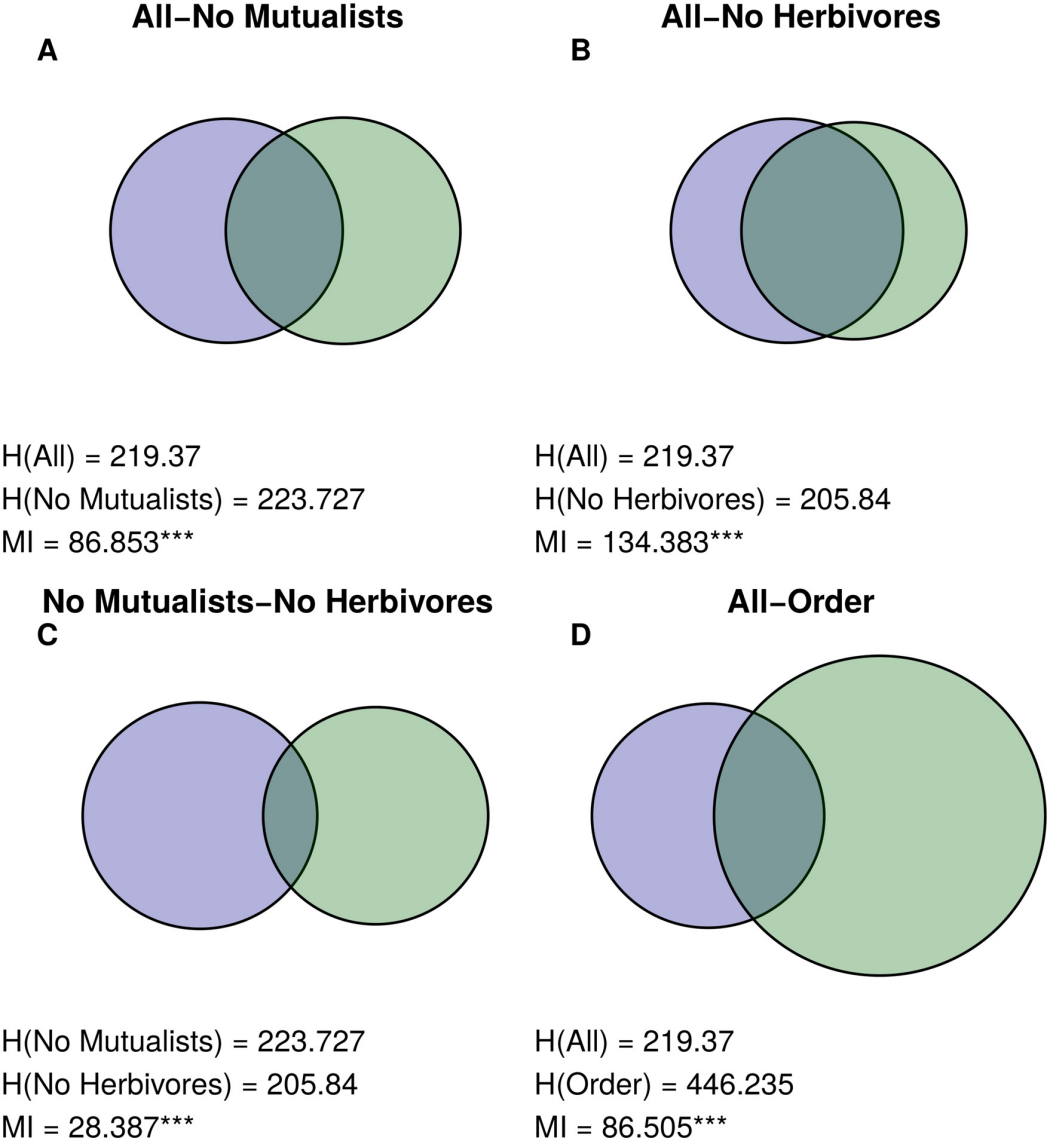
Similarities between Doñana Biological Reserve plant partitions. Venn Diagrams for similarity between pairs of plant partitions for the Doñana web: (A) complete and mutualist-removal webs, (B) complete and herbivore-removal webs, (C) mutualist-removal and herbivore-removal webs, and (D) complete network and taxonomic order. Figure structured as in Fig 3. This Figure includes only comparisons relevant to the main text; for all comparisons, see S4 Fig.

**Fig 7:**
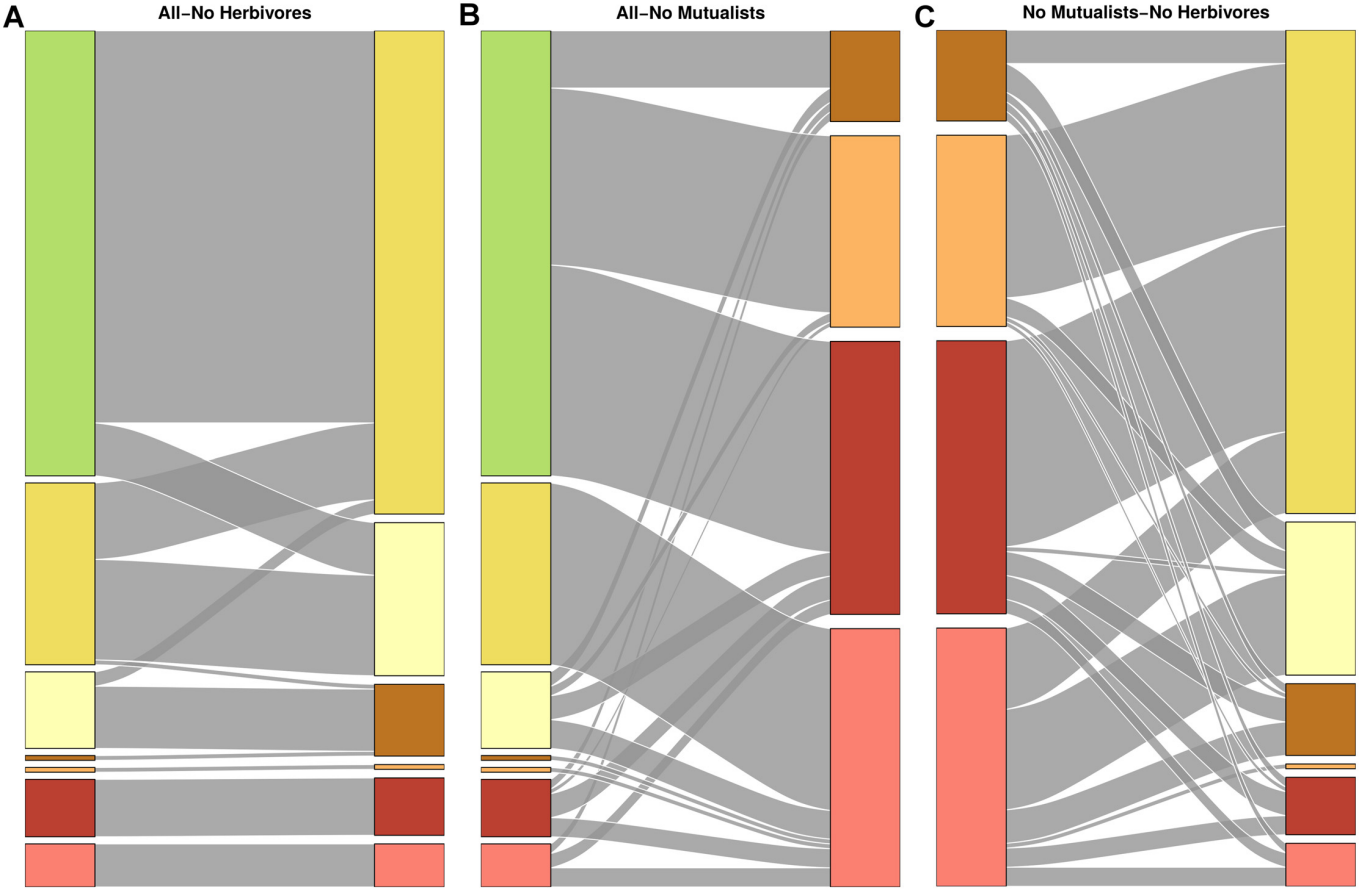
Similarity between Doñana plant groupings. Alluvial diagrams comparing the plant groupings for (A) complete and herbivore-removal webs, (B) complete and mutualist-removal webs, and (C) herbivore-removal and mutualist-removal webs. All three comparisons show major areas of similarity, but the groupings in (C) have many more conflicts than (A) and (B).

Plants in the complete Doñana network grouped in a similar way to both the herbivore-removal and mutualist-removal networks. The herbivore-removal and mutualist-removal partitions were much less similar to each other than to the complete partition, although still more similar than expected by chance (Figs 6 and 7). The herbivore-removal grouping contained much more information about the complete grouping than the mutualist-removal one did, possibly because mutualists greatly outnumbered herbivores in this network, both in number of species (207 and 14 species, respectively) and interactions with plants (576 and 221 interactions).

### Norwood Farm

**Fig 8:**
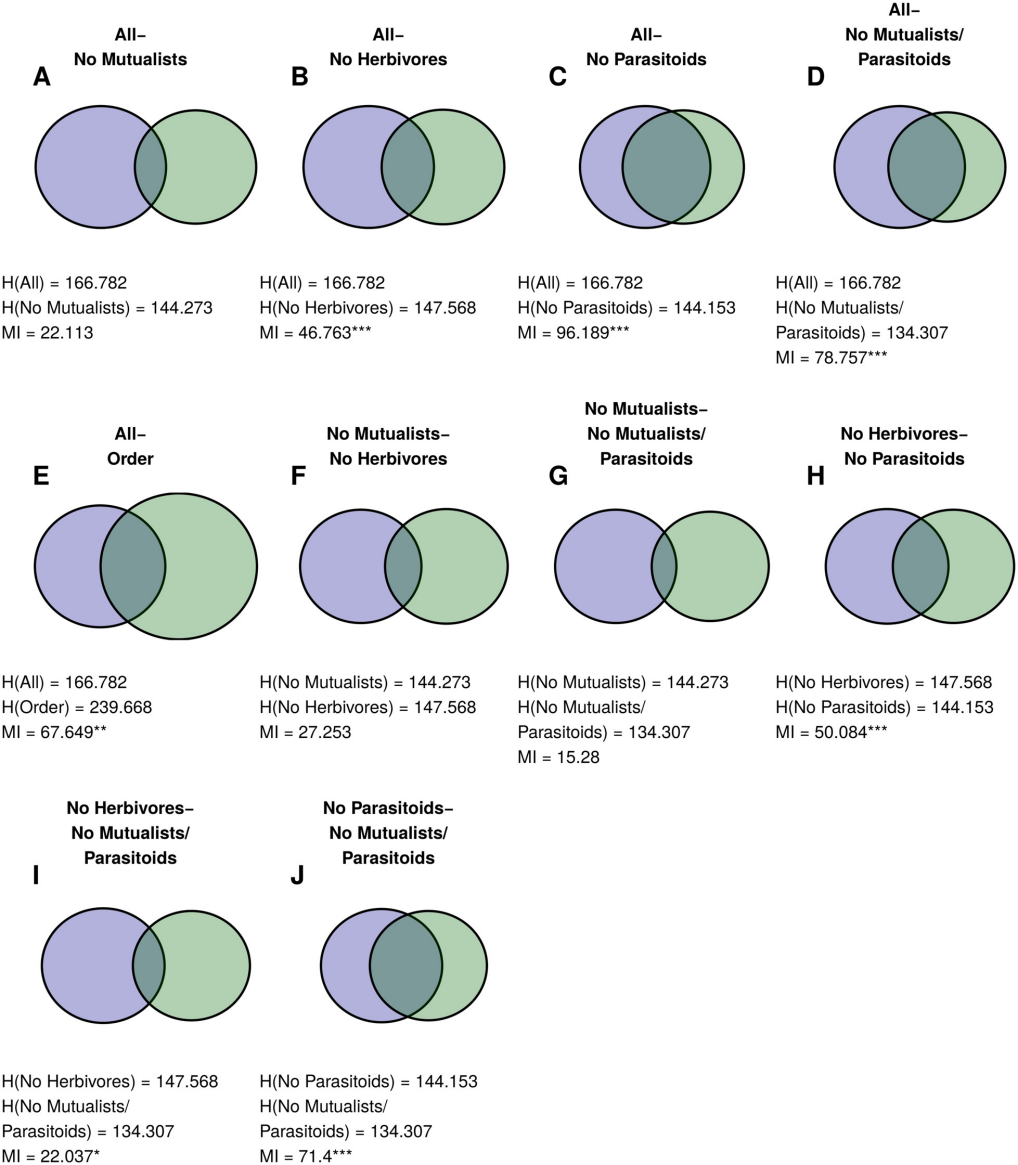
Similarities between Norwood Farm plant partitions. Venn Diagrams for similarity between pairs of plan partitions for the Norwood Farm webs: (A) complete mutualist-removal webs, (B) complete and herbivore-removal webs, (C) complete and parasitoid-removal webs, (D) complete and mutualist-and-parasitoid-removal webs, (E) complete web and taxonomic order, (F) mutualist-removal and herbivore-removal webs, (G) mutualist-removal and mutualist-and-parasitoid-removal webs, (H) herbivore-removal and parasitoid-removal webs, (I) herbivore-removal and mutualist-and-parasitoid-removal webs, and (J) parasitoid-removal and mutualist-and-parasitoid-removal webs. Figure structured as in Fig 3. Note that comparisons H-J are equivalent to the comparisons in Doñana, in that they show the effect of removing mutualists and herbivores in the absence of parasitoids. As in Figs 3 and 6, only partition comparisons relevant to the main text are included; for all comparisons, see S5 Fig.

**Fig 9:**
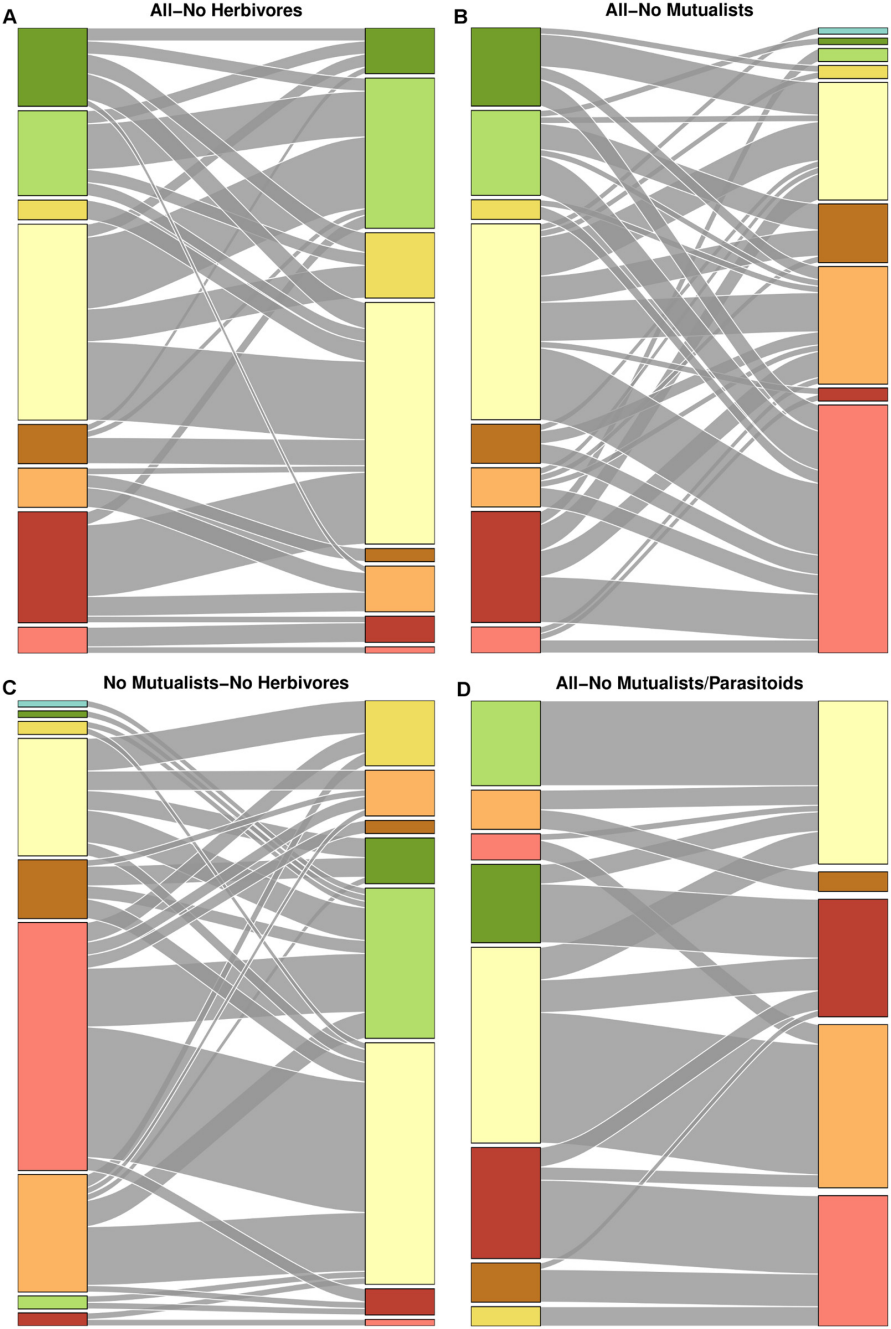
Similarity between Norwood plant groupings. Alluvial diagrams comparing the plant groupings for (A) complete and herbivore-removal webs, (B) complete and mutualist-removal webs, (C) herbivore-removal and mutualist-removal webs, and (D) complete and mutualist-and-parasitoid-removal webs. In general, these grouping are more dissimilar than seen in the Tatoosh and Doñana systems, and only (A) and (D) show more similarity than expected by chance.

When parasitoids were excluded from the network, results for the Norwood community were qualitatively similar to Doñana. Mutualist-removal and herbivore-removal groupings were similar to the grouping with both mutualists and herbivores (but not parasitoids), but were less similar to each other (Figs 8 and 9). Interestingly, removing herbivores in this network changed group structure more than removing mutualists, even though there were many more mutualists than herbivores (251 and 62 species), and mutualists and herbivores had almost exactly the same number of interactions with plants (569 and 570 interactions).

Including parasitoids in the network markedly changed the resulting group structure. The complete grouping remained similar to the herbivore-removal grouping (which also removes parasitoids, since they only interact with herbivores). However, the mutualist-removal partition was no more similar to the complete one than expected by chance. Surprisingly, the partition for the mutualist-parasitoid-removal was more similar to the complete partition than either the herbivore or mutualist removal groupings.

### Taxonomic Groupings

**Fig 10:**
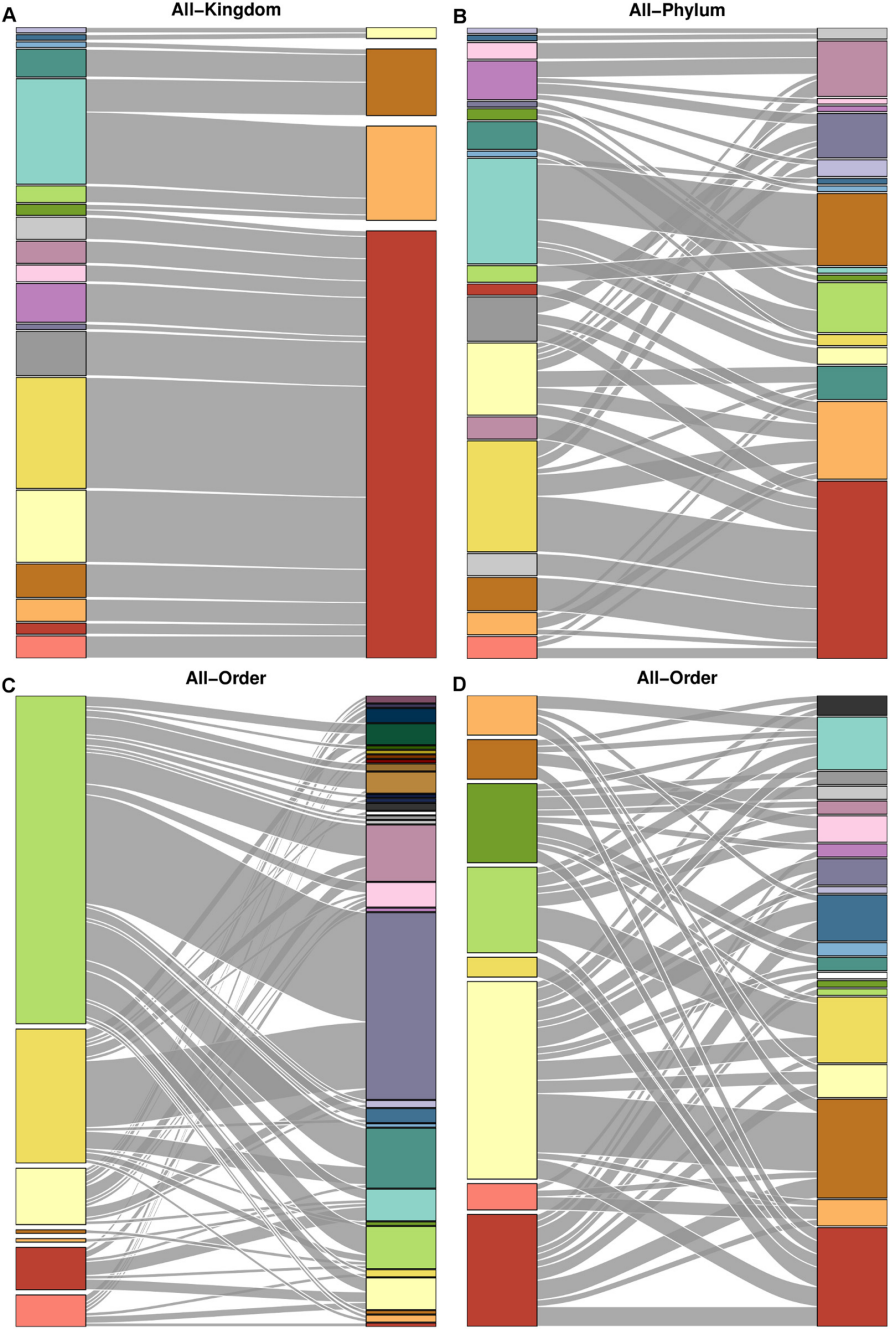
Comparison between complete and taxonomic groupings. Alluvial diagrams comparing complete web groupings with taxonomic groupings for (A) Tatoosh and kingdom, (B) Tatoosh and phylum, (C) Doñana and plant order, and (D) Norwood and plant order. All groupings are more similar than expected by chance. Kingdom matches very closely with the complete Tatoosh grouping, but has so few categories that it still provides very limited information. The other taxonomic groupings have more categories but still provide relatively little information.

Taxonomic grouping provided some information about complete groupings for all three networks. The Tatoosh complete grouping is almost perfectly nested within the species classification by kingdom (Figs 3 and 10). However, because this classification is so broad, it provides less information than phylum, even though the phylum grouping and complete grouping are dissimilar in many areas. In the Doñana and Norwood webs, taxonomic order was significantly similar to the complete groupings (Figs 6 and 8, respectively), but this similarity was not even across orders: some orders strongly grouped together in the complete groupings, while many others were scattered between several groups (Fig 10).

## Discussion

The extended group model is able to take large networks of great complexity, with many types of interactions, and condense them down to their essential structure. This results in a significant decrease in network complexity. It is able to reduce the Tatoosh intertidal network from 110 species down to 19 groups of ecologically equivalent taxa. Using a subset of these interaction types reduces the number of groups simply because the model has less information to work with, and indeed we see that the number of groupings in Tatoosh is greater with all interactions than with trophic interactions only (19 and 13 groups, respectively). Thus, using this extension of the group model in conjunction with interaction web information gives us a slightly more refined view of the network structure. It is notable that the Tatoosh groupings corresponded closely to many ecologically natural sets of species. The model does not use any ecological information outside of the network structure itself, but these patterns of interaction alone are enough to make highly specific distinctions, such as between limpets and other types of grazers.

As one possible use of the extended group model, we consider the effects of including or excluding interaction types from a network. In the Tatoosh network, removing interactions did not exclude species from the network, and even removing large numbers of interactions—nontrophic interactions constitute 54% of interactions in this system—had relatively little effect. This means that in these networks, species which have similar patterns of predation also have similar patterns of competition and mutualism, and so forth. In Doñana and Norwood, however, removing interaction types mean that entire classes of species were also included, and these removals had a comparatively large effect on the group structure. This suggests that plants which are similar to mutualists are not necessarily also similar to herbivores.

The grouping differences between these two network types could arise for many reasons. Sampling effects could play a role, since only three networks were available for study. Intrinsic differences between terrestrial and intertidal systems might also have an effect, since marine systems exhibit strong trophic control [5, 33]. Because terrestrial mutualists and herbivores are not as tightly linked by these top-down forces, plant groupings based on these different groups might not be tightly linked either. Another possibility relates to the biological traits which underly species interactions. In the intertidal, traits which are relevant to predators, such as mobility and presence of a shell, are likely also relevant for other types of interactions. For example, sessile species will tend to compete for space, and shelled species may benefit other species by providing shelter. In the Tatoosh community, mobile and sessile species rarely group together, and this is also true for shelled and shell-less species (Fig 5, S1 Table). In terrestrial plants, traits and structures that are relevant to mutualists (flowers, fruits) are relatively distinct from those that are relevant to herbivores (foliage, defense compounds). This specificity of traits relevant to particular interactions could decrease the group similarity when considering different parts of the network.

Taxonomic classification provides an obvious natural grouping for species. However, although taxonomic grouping provided some information about the complete group structure (as has been found for food webs in [34]), they were never the best way to estimate it. Taxonomic groupings were either too broad to provide much information, or grouped species differently than the complete network. This coincides with recent findings that phylogenetic relatedness poorly predicts interaction patterns and species roles in green algae [30, 35, 36].

The recursive definition of the group can lead to interesting outcomes. For example, parasites have a dramatic effect on Norwood group structure in the absence of mutualists. This is likely the result of a domino effect where parasitoids influence the grouping of herbivores, and herbivores influence the grouping of plants. Thus, when mutualists are removed, parasitoids have a major effect on the broad structure of the system. But in the presence of mutualists, plants are being influenced by both mutualists and herbivores, and the signal is lost. This result adds to the abundant evidence for the importance of including parasites in networks [37–40] (but see [41]), but more generally, it demonstrates that species need not be directly connected to influence each other. This situation reflects ecological reality, in that species may place evolutionary pressures on each other via a common species, which has been documented specifically between plant pollinators and plant herbivores [7, 42].

The extended group model may help us study and understand interaction networks in a variety of ways. One possible approach is simply to examine the grouping and look for surprises. For example, only crustose and coralline algae form a group separate from other algae based on trophic information in the Tatoosh network, but when nontrophic information is also incorporated, several kelp species form an additional distinct group. This suggests that these two groups interact differently in the network, in a way that specifically relates to their nontrophic interactions. On closer examination of the network structure, this difference is likely related to the fact that these kelps have a negative effect on the growth of the other algal group, but the other algae do not negatively affect the kelps.

Similarly, because the group model identifies ecologically equivalent species, it can be used to identify species which are performing unique roles in the community. In the Tatoosh network, there are three species which are not grouped with any others: detritus, diatoms, and *Anthopleura elegantissima*, a sea anemone. Detritus and diatoms are both relatively unique food sources that are present in the water column, rather than attaching to the rock. It is, perhaps, less obvious why anemones are so unique as to be placed in their own group. However, they are unlike all other species in the system in that they are predatory but sessile, unlike other predators which move to find and consume their prey. *Anthopleura* also has endosymbiotic algae which are implicitly included in the network through the anemone’s interactions. Although the existence of a group does not guarantee that it is essential for ecosystem functioning, looking at groups with one or few species may be a useful way to identify species which play unique roles and whose removal might have a larger effect on the system, since no other species are able to take their place.

Another possible application of the group model is to have a simpler version of the network to work with. These simplified networks are easier to take in and comprehend by eye. They may also be useful for finding generalities across networks. This is currently difficult to do, since there are few interaction networks currently available. In the future, it would be interesting to see if communities tend to form similar numbers of groups, if specific species always perform unique roles, if similar groups tend to form at specific trophic levels, and so forth. Since species within a group perform similar roles in the community, we speculate that these species might exhibit similar population dynamics. It is possible that simplifying networks down to their group structure could be a useful way to simplify multi-species dynamical models.

### Conclusions

The extended group model is a general method for identifying functionally equivalent nodes in signed directed networks. We have discussed the method as applied to ecological interaction webs, but the methodology could also be used to study the structure of networks of gene regulation [43, 44], sensors [45], and even social networks which incorporate both positive and negative social interactions [46]. The generality of the method does not detract from its usefulness in ecology; in fact, the model is able to identify highly specific ecological roles. This model is a new and useful exploratory tool to understand and compare the coarse-grained structure of ecological communities.

## Supporting Information

S1 Text Supplementary methodological information. Supplementary information about networks, taxonomic data, search algorithm, and model comparisons.
(PDF)

S1 Fig Optimal grouping structure for the Tatoosh Island mussel bed trophic network. Figure structured as in main text Fig 1. Numbered list of taxon names is given in S1 Table.
(EPS)

S2 Fig Optimal grouping structure for the Tatoosh Island mussel bed nontrophic network. Figure structured as in main text Fig 1. Numbered list of taxon names is given in S1 Table.
(EPS)

S3 Fig Similarities between Tatoosh Mussel Bed partitions. Table structured as in main text Fig 3, but containing all pairwise comparisons between partitions for the Tatoosh Mussel Bed.
(EPS)

S4 Fig Similarities between Doñana Biological Reserve plant partitions. Table structured as in main text Fig 6, but containing all pairwise comparisons between partitions for Doñana Biological Reserve.
(EPS)

S5 Fig Similarities between Norwood Farm plant partitions. Table structured as in main text Fig 8, but containing all pairwise comparisons between plant partitions for Norwood Farm.
(EPS)

S1 Table Group identities for Tatoosh mussel bed species. Species in the Tatoosh mussel bed network are listed in order of grouping in the complete network as shown from left to right in main text Fig 5, for the trophic network as in S1 Fig, and for the nontrophic network as in S2 Fig.
(PDF)
