# Supplementary material for: What Can Interaction Webs Tell Us About Species Roles?
Source: PLoS Comput Biol. 2015 Jul 21;11(7):e1004330. doi: 10.1371/journal.pcbi.1004330 (PMC4511233; doi:10.1371/journal.pcbi.1004330)
Supplement: S1 Text — Supplementary information about networks, taxonomic data, search algorithm, and model comparisons. (PDF) [file pcbi.1004330.s001.pdf]

Supplementary Information

Supplemental Network Information

In the Norwood farm network [1], indirect interactions (i.e., parasitoids which indirectly benefitted plants by parasitizing herbivores) were excluded from the adjacency matrix. In [1], leaf-miner parasitoids were assumed to be a reasonable proxy for the interactions between plants and leaf-miners. However, since direct leaf-miner network data were not available, we chose to exclude these species altogether. Two species in this system interacted in the network in multiple ways (interacting with some species as a plant mutualist and with others as an herbivore, for example). Each of these species was listed as a single node in the adjacency matrix, but both interaction types were kept. In these cases, when species of one interaction type were removed, only the links of that interaction type were removed.

In the Doñana and Norwood webs, some plant species became disconnected from the large cluster when mutualists or herbivores were removed. However, these species were not excluded from the group model selection process, to make the single-interaction versions of the networks more directly comparable to the complete versions.

Taxonomic Data

Taxonomic data were obtained from the ITIS website at `//www.itis.gov/downloads/index.html` and resolved to the species, genus, or family level, whichever was the most detailed that could be obtained from this database. Taxa which could not be identified through the ITIS database were identified by hand to the highest resolution possible using information from the Encyclopedia of Life [2], and outdated names were changed by hand where possible. Nodes that were not resolved to at least the phylum (for the Tatoosh network) or order (for the Doñana and Norwood networks) level were excluded from the taxonomy-based grouping and similarity calculations between partitions.

Search Algorithm

The space of possible groupings was explored using Metropolis-coupled Markov Chain Monte Carlo (MC^3), also known as parallel tempering [3, 4]. This search strategy allows multiple MCMC chains at different temperatures to run in parallel, occasionally giving chains the opportunity to swap temperatures. Here we use the term 'temperature' as it is used in simulated annealing, where the probability of accepting a bad move is a function of the temperature. We allowed our chains to take temperatures which ranged from the hottest temperature of slightly above 0 (all proposed steps accepted with equal probability) to the coldest temperature, 1 (proposed steps accepted with probability directly proportional to their likelihood). Every 20 steps, we allowed chains the opportunity to swap temperatures. These swaps proceeded in a "bucket brigade" fashion [5], such that each chain had the opportunity to swap temperatures with adjacent temperatures from hottest to coldest with probability

$$min\left[1, exp\left(\left(\frac{1}{t_i} - \frac{1}{t_{i+1}}\right)\left(log\left(P(N|G_i)\right) - P(N|G_{i+1})\right)\right)\right] \tag{1}$$

where t_i is the temperature of chain i, and temperatures are arranged such that t_i is hotter than t_{i+1}. In this way, the hottest chain may cool to become the coldest chain in a single round of swaps if it happened to find a promising part of the solution space.

Case studies have suggested that the algorithm performs best when the probability of accepting a swap is close to 20% [5]. Based on this and our own preliminary study, we calibrated the number of chains for each network to result in a swap acceptance rate between 15 and 35 percent. The swap acceptance probability for a given number of chains tended to decrease with network size, so larger networks were generally given more chains. Temperatures were uniformly spaced, as preliminary testing indicated that

alternate spacing schemes (*e.g.*, logarithmic) resulted in very high swap acceptance rates and inferior groupings.

Restricted Growth Function (RGF)

Groupings were stored as a vector of length equal to the number of species S. The ith element of this vector held the group membership of species i. Groups were given a number between 0 and $(S-1)$. After initializing the starting solution for each chain, and after each mutation step, the solution was run through the RGF algorithm. This algorithm renumbers the groups such that as you move from left to right through the vector, new groups are numbered sequentially. For example, the solution [2 3 1 1 4 2] would be represented as [0 1 2 2 3 0] after being run through the RGF. The renumbering does not change the solution, since the group labelling is arbitrary, but it ensures that each solution has a single consistent representation. This algorithm runs in linear time and improves the efficiency of the parallel tempering algorithm, since it spends less time rediscovering solutions that are identical but encoded differently.

Gibbs Sampler

The mutation step for each chain used a Gibbs sampler, which ran as follows: A single species was chosen for mutation. Then, the algorithm calculated the Bayes factor for each potential group assignment for that species, including its current group assignment and assigning it the species its own unique group. The new group assignment was chosen based on probabilities B_i^T, where B_i is the Bayes factor of the ith group, and T is the temperature of the chain.

Jackknife Resampling

An implicit assumption of the group model as used in this study is that the "complete" network structure, containing all available interaction data, is the "true" network structure. That is, the model assumes that there are no missing or erroneous interactions. There are many possible ways to test the robustness of the method to incomplete data. Jackknife resampling was chosen due to the relative simplicity of performing and interpreting the results. Resampling was only performed using the Tatoosh Island network, because this was the only network with fast enough convergence to be computationally feasible. Species were removed one at a time from the complete network, then were partitioned using the group model 25 times, with the algorithm running for 10000 steps with 20 chains each run. The 25 runs converged to between 2 and 6 solutions with similar marginal likelihoods, suggesting that the algorithm was able to adequately explore the solution space. The best grouping for each species removal was compared to the best grouping before species removal. The match between the grouping before and after removal was calculated as $\frac{MI}{MI_{max}}$, that is, the observed MI over the maximum MI possible for the two partitions (MI_{max} is equivalent to the minimum of the two entropies).

Null Model for Partition Comparison

There are multiple possible null models when comparing the observed mutual information to what is expected by chance. An intuitive null model for species grouping would be to assign each species a group identity from 1 to S. However, this allows the null to have more or fewer groups than observed, which changes the number of parameters in the model. A similar problem exists if we choose to assign each species a group identity from 1 to g (the number of groups in the observed partition). The null will have the same number of groups as observed, but will likely have different numbers of species in each group. the number of species in the different groups has a large effect on the entropy, which provides an upper bound on the MI. Therefore, we choose to randomize the grouping by shuffling the species, such that the number of species in each group remains identical to the observed partition, but the species identities

in the groups are randomized. This procedure maintains the entropies of each partition, so that the MI has the same upper bound, but allows us to get a distribution of what MI would be expected by chance, in the absence of the effect of network structure. This is the most conservative null model of the three.

References

1. Pocock MJO, Evans DM, Memmott J. The robustness and restoration of a network of ecological networks. Science. 2012 Feb;335(6071):973–7. Available from: http://www.ncbi.nlm.nih.gov/pubmed/22363009.

2. Encyclopedia of Life;. Available from: http://www.eol.org.

3. Geyer CJ. Markov chain Monte Carlo maximum likelihood. In: Keramidas, editor. Computing Science and Statistics: Proceedings of the 23rd Symposium on the Interface. Fairfax Station: Interface Foundation; 1991. p. 156–163.

4. Altekar G, Dwarkadas S, Huelsenbeck JP, Ronquist F. Parallel Metropolis coupled Markov chain Monte Carlo for Bayesian phylogenetic inference. Bioinformatics (Oxford, England). 2004 Feb;20(3):407–415. Available from: http://www.ncbi.nlm.nih.gov/pubmed/14960467.

5. Earl DJ, Deem MW. Parallel tempering: theory, applications, and new perspectives. Physical chemistry chemical physics. 2005 Dec;7(23):3910–6. Available from: http://www.ncbi.nlm.nih.gov/pubmed/19810318.
