# Supplementary material for: What Can Interaction Webs Tell Us About Species Roles?
Source: PLoS Comput Biol. 2015 Jul 21;11(7):e1004330. doi: 10.1371/journal.pcbi.1004330 (PMC4511233; doi:10.1371/journal.pcbi.1004330)
Supplement: S1 Table — Species in the Tatoosh mussel bed network are listed in order of grouping in the complete network as shown from left to right in main text Fig 5, for the trophic network as in S1 Fig, and for the nontrophic network as in S2 Fig. (PDF) [file pcbi.1004330.s007.pdf]

Species Names	All	Trophic	NonTrophic
Particulate Detritus	1	6	3
Phytoplankton	1	6	12
Detritus	2	6	1
Larus glaucescens	3	4	2
Corvus caurinus	3	4	2
Haematopus bachmani	3	5	2
Dialula (Discodoris) sandiegensis	4	10	3
Archidoris montereyensis	4	10	3
Odostomia columbiana	4	10	3
Fissurelidia (Megatebennus) bimaculatus	4	10	3
Hermissenda (Phidiana) crassicornis	4	10	3
Opalia montereyensis	4	10	3
Haliaeetus leucocephalus	4	10	3
Falco peregrinus	4	10	3
Pelagic Fish	4	10	3
Pinnotheres plsum	4	10	5
Amphissa columbiana	4	11	3
Cirolana	4	11	3
Calliostoma ligatum	4	11	3
Ophiuroidea	4	11	3
Alia	4	11	3
Staphylinid Beetle	4	11	3
Small Turrid	4	11	3
Henricia	4	11	3
Chironomidae	4	11	5
sand tube worm	4	11	5
Zooplankton	4	11	5
Emplectonema gracile	5	5	3
Leptasterias hexactis	5	5	3
Notoplana	5	5	3
Pisaster ochraceus	5	5	3
Oligocottus maculosus	5	5	5
Clinocottus embryum	5	5	5
Clinocottus globiceps	5	5	5
Paranemertes peregrina	6	10	3
Amphiporus bimaculatus	6	10	3
Nereis vexillosa	6	11	3
Tonicella lineata	6	11	3
Phascolosoma agassizii	6	11	3
Cucumaria pseudocurata	6	11	3
Petrolisthes	6	11	3
Nereis sp.	6	11	3
Oedignathus inermis	6	11	3
Ceratostoma foliatum	6	11	3
Halosydna brevisetosa	6	11	3
Stichaeidae	6	11	3
Pagurid	6	11	4
Amphipoda	7	1	3

Idotea wosnesenskii	7	1	3
Chlorostoma (Tegula) funebralis	7	1	3
Katharina tunicata	7	1	5
Mopalia	7	1	5
Strongylocentrotus purpuratus	7	1	5
Strongylocentrotus droebachensis	7	1	5
Strongylocentrotus franciscanus	7	1	5
Littorina sitkana	8	12	3
Littorina scutulata	8	12	3
Lacuna vincta	8	12	3
Siphonaria thersites	8	12	3
Onchidella borealis	8	12	4
Lepidochitona	8	12	5
Lottia pelta	9	2	5
Lottia digitalis	9	2	5
Lottia paradigitalis	9	2	5
Lottia (Tectura) scutum	9	2	5
Ocenebra interfossa	10	3	3
Nucella ostrina	10	3	5
Nucella canalicalata	10	3	5
Nucella lamellosa	10	3	5
Chthamalus dalli	11	11	6
Balanus nubilus	11	13	5
Entodesma saxicola	11	13	5
Serpula vermicularis	11	13	5
Anthopleura	12	5	6
Sacharina (Hedophyllum) sessilis	13	8	7
Laminaria	13	8	7
Alaria	13	8	7
Costaria costata	13	8	7
Postelsia palmaeformis	13	8	7
Halichondria Haliclona	14	9	12
Phyllospadix	14	9	13
Diatoms	15	7	8
Corallina vancouvriensis	16	9	9
Articulated corralines	16	9	9
Petrocelis Ralfsia	16	10	8
Halosaccion glandiforme	17	8	10
Mastocarpus (Gigartina)	17	8	10
Endocladia muricata	17	8	10
Ulva	17	8	10
Porphyra	17	8	10
Acrosiphonia (Spongomorpha) coalita	17	8	10
Fucus distichus	17	8	10
Microcladia borealis	17	8	10
Iridaea	17	8	10
Leathesia marine (difformis)	17	8	10
Polysiphonia	17	8	10
Neorhodomela	17	8	10

Prionitis	17	8	10
Petalonia fasciata	17	8	10
Callithamnion pikeanum	17	8	10
Scytosiphon lomentaria	17	8	10
Cumagloia andersonii	17	8	10
Analipus japonicus	17	8	10
Enteromorpha	17	8	10
Semibalanus cariosus	18	13	11
Balanus glandula	18	13	11
Mytilus trossulus	19	13	12
Pollicipes polymerus	19	13	12
Eudistylia	19	13	12
Mytilus californianus	19	13	13

Table 1: **Group identities for Tatoosh mussel bed species.** Species in the Tatoosh mussel bed network are listed in order of grouping in the complete network as shown in main text Fig. 2. Group identities for the trophic and nontrophic networks (as in Fig. S1 and S2, respectively) are also listed.
